# Spatio-temporal analysis of the relationship between climate and hand, foot, and mouth disease in Shandong province, China, 2008–2012

**DOI:** 10.1186/s12879-015-0901-4

**Published:** 2015-03-24

**Authors:** Yunxia Liu, Xianjun Wang, Chunkun Pang, Zhongshang Yuan, Hongkai Li, Fuzhong Xue

**Affiliations:** Department of Epidemiology and Biostatistics, School of Public Health, Shandong University, Jinan, Shandong China; Shandong Center for Disease Control and Prevention, Jinan, Shandong China; Institute office, Shandong Academy of Medical Science, Jinan, Shandong, China

**Keywords:** Hand, foot and mouth disease (HFMD), Spatial epidemiology, Bayesian approach, Climatic indicator, China

## Abstract

**Background:**

Hand, foot, and mouth disease (HFMD) is the most common communicable disease in China. Shandong Province is one of the most seriously affected areas. The distribution of HFMD had spatial heterogeneity and seasonal characteristic in this setting. The aim of this study was to explore the associations between climate and HFMD by a Bayesian approach from spatio-temporal interactions perspective.

**Methods:**

The HFMD data of Shandong Province during 2008–2012 were derived from the China National Disease Surveillance Reporting and Management System. And six climatic indicators were obtained from the Meteorological Bureau of Shandong Province. The global spatial autocorrelation statistic (Moran’s *I*) was used to detect the spatial autocorrelation of HFMD cases in each year. The optimal one among four Bayesian models was further adopted to estimate the relative risk of the occurrence of HFMD via Markov chain Monte Carlo.

**Results:**

The annual average incidence rate of HFMD was 104.40 per 100,000 in Shandong Province. Positive spatial autocorrelation appeared at county level (Moran’s *I* ≥0.30, *P* < 0.001). The best fitting Spatio-temporal interactive model showed that annual average temperature, annual average pressure, annual average relative humidity, annual average wind speed and annual sunshine hours were significantly positive related to the occurrence of HFMD. The estimated relative risk of 36, 87, 91, 79, 65 out of 140 counties for 2008–2012 respectively were significantly more than 1.

**Conclusions:**

There were obvious spatio-temporal heterogeneity of HFMD in Shandong Province, and the climatic indicators were associated with the epidemic of HFMD. Bayesian approach should be recommended to capture the spatial-temporal pattern of HFMD.

## Background

Hand, foot, and mouth disease (HFMD) is a common communicable disease usually affecting children, particularly those aged 5 years and younger [1]. It is most frequently caused by Coxsackievirus A16 and Enterovirus 71 [1,2], and is often characterized by a distinct clinical presentation of fever, or vesicular exanthema on their hands, feet, mouths, or buttocks [3,4].

Over the last decades, many large-scale outbreaks of HFMD were reported in East and Southeast Asia and have caused major public health concerns worldwide, especially in the affected countries [5-8]. In China, several outbreaks have also been reported, such as Linyi in Shandong (2007) [9], Fuyang in Anhui (2008) [10], Shanghai (2009) [11], Nanchang in Jiangxi (2010) [12], etc. Since its beginning in Fuyang, large-scale epidemic have resulted in the deaths of many children, HFMD was classified as a class C notifiable infectious disease by the Ministry of Health in China on 2 May 2008 [13]. To date, HFMD is still one of the leading causes of child death and a serious public health issue in China.

Currently, there are still no available effective vaccines or antiviral treatments specifically for HFMD. Thus, it is quite important to identify the possible risk factors for HFMD. Many epidemiological studies have been conducted to explore individual-level risk factors (e.g., demographic factors, socio-economic determinants and behavioral factors) [14-20]. Although these individual factors may play a role in HFMD incidence, group-level factors are more important for public health intervention. Considering the spatial distribution characteristics of HFMD, several researches (i.e., ecological studies) have been conducted at group-level (e.g., county, community, or city) based on spatial analysis methods (e.g., spatial regression model and spatial paneled model), which was helpful to understand the emerging trend of HFMD in certain area and explore the ecological causes of disease incidence [21], and can guide us to take the correct and timely public health interventions to prevent the outbreak. Among these, some studies found that the incidence of HFMD was associated with some climatic indicators (e.g., average temperature, relative humidity and annual sunshine hours) [22-26]. Therefore, the effects of climatic indicators on HFMD should be paid more attention on, especially in the context of climate change. In addition, the occurrence of HFMD presents significant seasonality, i.e., temporal characteristic [23,27-31]. Therefore, spatio-temporal model should be preferred, which can not only help to recognize the spatial and temporal trend of disease, but also make prediction and guide us to formulate and implement appropriate regional public health intervention strategies to prevent and control this disease.

However, few studies from spatio-temporal interactions perspective, have attempted to explore this relationship and to assess how climate affect HFMD incidence. On the other hand, Bayesian methods can take into account possible correlations and covariates’ effects and fully utilize the related information of disease and prior knowledge, it has been extensively used in many studies and has been recognized as a powerful means to provide more robust estimates [32-35].

Thus, the goal of this study is to investigate the relationships between HFMD incidence and climatic indicators based on Bayesian approaches. Using the data of new cases of HFMD reported during 2008–2012 in Shandong Province, four Bayesian models were constructed and compared, the best fitting one was further adopted to estimate the effects of six key climatic indicators, with the aim of increasing our knowledge of the true underlying geographic distribution of HFMD rates.

## Method

### Study area

**Figure 1:**
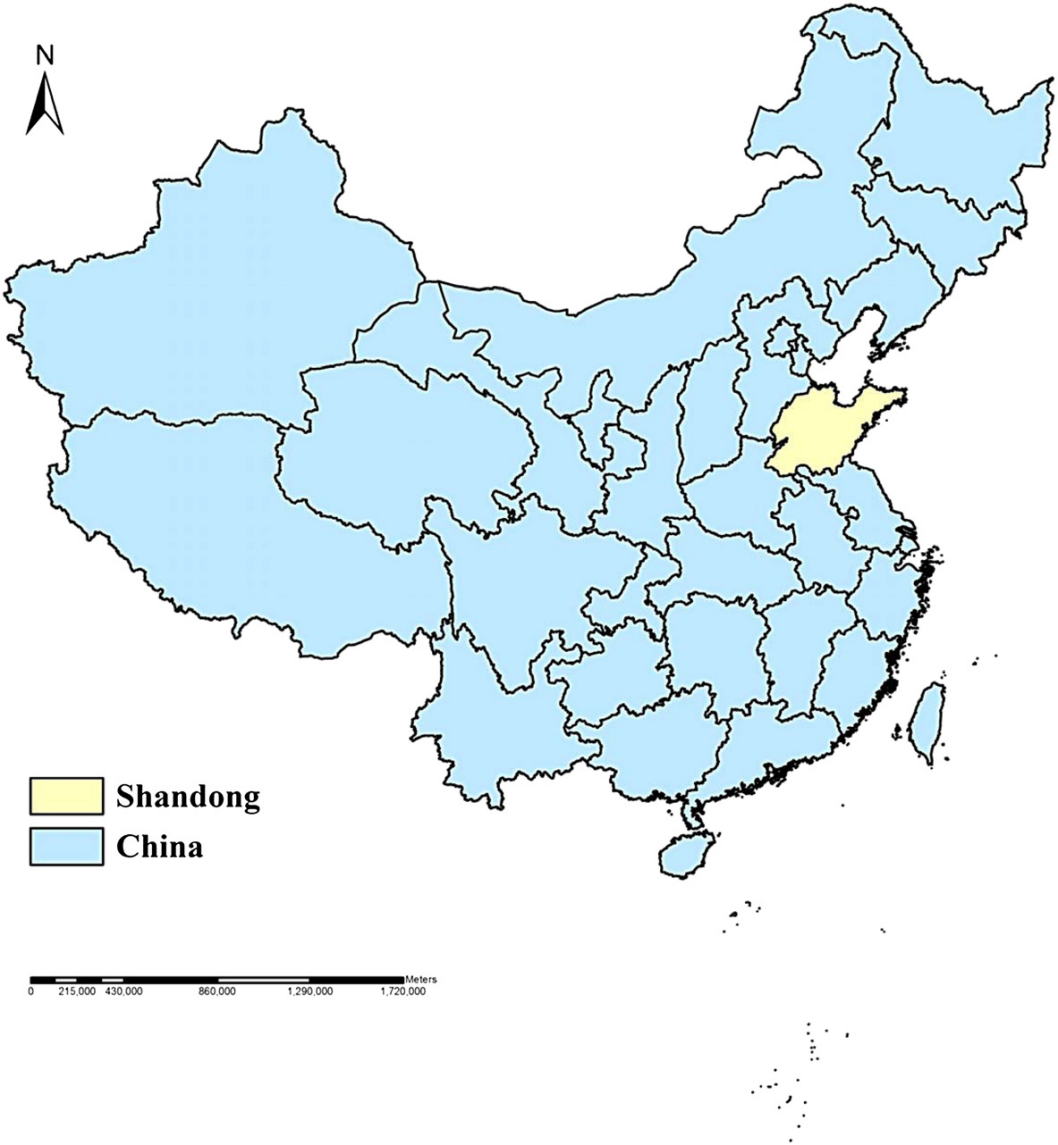
**The location of study area, Shandong Province in China.**

We performed this ecological spatial study of HFMD in Shandong Province (Figure 1), a coastal province in Eastern China with a population of approximately 97.33 million in 2013 [36]. Its area of 156,700 square kilometers was divided into 17 municipal districts which including 140 subdistricts (counties). Considering the county administrative level was often used for the HFMD-decision making process in China [27], county was used as the spatial unit of analysis (Figure 1).

### HFMD data

Data concerning to the reported cases of HFMD were provided by the Shandong Center for Disease Control and Prevention collected from the China Information System for Disease Control and Prevention (CISDCP), which included information about sex, age, occupation, and regional distribution. The diagnosis of HFMD was according to the clinical criteria from the HFMD Control and Prevention Guide offered by the Chinese Ministry of Health [37]. The report cards of HFMD cases filled out by professional doctors were collected by trained reporter and then input into the CISDCP within 24 hours based on the P. R. China infectious disease prevention and cure statute [27]. The data collected in this paper contained the basic demographic and incidence information for 497,876 HFMD cases during 2008 to 2012 in Shandong Province.

### Demographic and climatic data

The climatic indicators included annual average temperature, annual average pressure, annual average relative humidity, annual average wind speed, annual sunshine hours and annual precipitation, which were obtained from the Meteorological Bureau of Shandong Province. The corresponding demographic data of each county over the study period referred to Shandong Statistical Yearbook [38-42].

### Ethics statement

HFMD data were provided by Shandong CDC extracted from annual reports, which provided summarized count data of patients reported by county and month. All data were anonymized.

### Statistical analysis

The frequencies of HFMD were summarized monthly and annually by geographic area (i.e., county), and the incidence rate of HFMD (per 100,000 population) in each county was calculated by HFMD counts divided by the corresponding population. We firstly analyzed the monthly temporal distribution characteristic of HFMD over the study period. The autocorrelation statistic (Moran’s *I*) [43] was then used to detect the global spatial autocorrelation of HFMD cases. The software GeoDa™ 0.9.5-i was used to conduct the analysis.

Considering the epidemic characteristics of HFMD, a Bayesian approach [32-35,44] was utilized to explore the relationship between HFMD and climatic indicators. For each county *i*(*i* = 1, 2, ⋯, *n*) and each year *t*, the expected number of HFMD cases (*ε*_*it*_) was estimated as the provincial overall mean rate in the year *t* multiplied by the population of the county (*ε*_*it*_ = *meanrate*_*t*_ × *pop*_*it*_). The relative risk (*RR*) *λ*_*it*_ was calculated as the observed number of HFMD cases divided by the expected. The observed number of cases (*y*_*it*_) in the *n* counties could be treated as one realization of Poisson random variables, with means *μ*_*it*_, i.e., *y*_*it*_ ~ *Pois*(*μ*_*it*_), where *μ*_*it*_ = *ε*_*it*_ × *λ*_*it*_. *λ*_*it*_ is a function of the effects of *k* covariates (*x*_*kit*_) as well as spatial and temporal random effects.

We constructed four Bayesian models with climatic variables. The first model only included non-spatial random effects (Non-spatial model), *λ*_*it*_ = exp(*β*_0_ + *β*_1_*x*_1*it*_ + *β*_2_*x*_2*it*_ + *β*_3_*x*_3*it*_ + *β*_4_*x*_4*it*_ + *β*_5_*x*_5*it*_ + *β*_6_*x*_6*it*_ + *v*_*i*_). Where *β*_0_ represented the mean incidence over all counties and time periods with a flat distribution; *β*_1_, *β*_2_,…, *β*_6_ were the coefficients of climatic variables following non-informative Gaussian priors with zero mean and precision equal to 10^−5^. A non-spatially structured random effect *ν*_*i*_ was included to account for extra-Poisson variation due to non-measured important covariates, with independent Gaussian distribution having zero mean and variance ${\sigma}_v^2$. We defined neighborhood as adjacent counties with simple binary adjacency weights, i.e., *w*_*ij*_ = 1 if area *i* and *j* share a common boundary and *w*_*ij*_ = 0 otherwise [44]. The second model considered spatial and non-spatial random effects (Spatial model), *λ*_*it*_ = exp(*β*_0_ + *β*_1_*x*_1*it*_ + *β*_2_*x*_2*it*_ + *β*_3_*x*_3*it*_ + *β*_4_*x*_4*it*_ + *β*_5_*x*_5*it*_ + *β*_6_*x*_6*it*_ + *v*_*i*_ + *θ*_*i*_). Where *θ*_*i*_ represented the spatially structured random effects, which account for the spatial dependence with the prior distribution taken as a conditional intrinsic Gaussian autoregressive model. In the third model, spatial, non-spatial and temporal random effects were included (Spatio-temporal model), *λ*_*it*_ = exp(*β*_0_ + *β*_1_*x*_1*it*_ + *β*_2_*x*_2*it*_ + *β*_3_*x*_3*it*_ + *β*_4_*x*_4*it*_ + *β*_5_*x*_5*it*_ + *β*_6_*x*_6*it*_ + *v*_*i*_ + *θ*_*i*_ + *ω*_*t*_). Where *ω*_*t*_ was a random term representing between-year variation and was assumed to be an autoregressive process. This model assumed spatial correlation between the counties was independent of the year. The fourth model added spatio-temporal interactive effects on the basis of spatio-temporal model (Spatio-temporal interactive model), *λ*_*it*_ = exp(*β*_0_ + *β*_1_*x*_1*it*_ + *β*_2_*x*_2*it*_ + *β*_3_*x*_3*it*_ + *β*_4_*x*_4*it*_ + *β*_5_*x*_5*it*_ + *β*_6_*x*_6*it*_ + *v*_*i*_ + *θ*_*i*_ + *ω*_*i*_ + *psi*_*it*_). This model included a different set of random spatial effects for each year following a conditional autoregressive model.

We adopted Markov chain Monte Carlo method to estimate the parameters by the public domain software package WinBUGS 1.4.3 software (Imperial College and MRC, London, UK). Totally 100,000 samples for each parameter of interested were generated, with a burn-in of 20,000 iterations to avoid the influence of the initial values. The convergence process was evaluated based on whether iteration trace and iteration history becoming stable, autocorrelation function being close to zero quickly. The Deviance Information Criterion (DIC) was used to assess the goodness-of –fit of models, smaller values of DIC indicated a more appropriate model [25].

## Results

### Prevalence of HFMD

From January 1, 2008 to December 31, 2012, there were 497,876 cases of HFMD reported in Shandong Province representing an average rate of 104.40 per 100,000. The rates at county level ranged between 18.65 and 328.44 per 100,000. Table 1 showed the detailed demographic characteristics of HFMD cases. Of the 497,876 cases, a majority of patients (473,183; 95.04%) were children younger than 6 years, the left (24,693; 4.96%) belonged to other age groups; 312,791 were male patients and 185,085 were female patients, with an average male-to-female ratio 1.69:1. Also it can be seen that most of HFMD cases were preschoolers (71.38% scattered children and 25.48% nursery children), the rest (3.14%) were students and others.

**Table 1:** **Demographic characteristics of HFMD cases in Shandong Province, 2008-2012**

	**2008**	**2009**	**2010**	**2011**	**2012**	**Total**
**Age**						
0~	5735	22983	18790	12272	10588	70368
1~	9447	43615	39586	25753	24090	142491
2~	7028	29746	31852	19636	18013	106275
3~	5244	19746	24920	17637	15978	83525
4~	2605	11724	13624	9954	10077	47984
5~	1245	5034	6586	5039	4636	22540
6~	617	2355	2436	2430	2088	9926
7~	316	1130	1053	995	1149	4643
8~	167	830	640	596	552	2785
9~	144	589	490	379	407	2009
10 ~ 14	285	1043	875	703	734	3640
≥15	148	362	423	342	415	1690
**Sex**						
Male	21213	86928	89302	59731	55617	312791
Female	11768	52229	51973	36005	33110	185085
Ratio of Male-to-Female	1.80	1.66	1.72	1.66	1.68	1.69
**Occupation**						
scattered children	22608	105590	99463	66004	61715	355380
nursery children	9119	29259	38167	26619	23698	126862
other	1254	4308	3645	3113	3314	15634
**Total**	32981	139157	141275	95736	88727	497876

**Figure 2:**
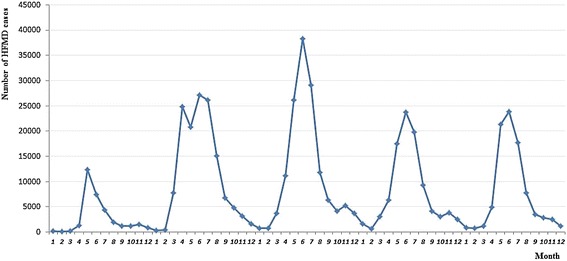
**Monthly distribution of HFMD cases in Shandong Province, 2008–2012.**

Figure 2 displayed the monthly distribution of HFMD cases during the study period, which presented significant seasonality. Obviously, the incidence peak appeared between April and August, which accounted for 83.00% of all reported cases.

### Spatial autocorrelation of HFMD cases

The results of the spatial autocorrelation test were listed in Table 2, demonstrating that high global spatial autocorrelation of HFMD existed at county level in Shandong Province within each epidemic year during 2008 to 2012 (Moran’s *I* ≥0.30, *P* < 0.001).

**Table 2:** **Results of the spatial autocorrelation test on HFMD cases in Shandong Province, 2008-2012**

**Year**	**Moran’s** ***I***	***Z*** **Score**	***P*** **-value**
**2008**	0.30	6.03	<0.001
**2009**	0.37	7.20	<0.001
**2010**	0.30	5.45	<0.001
**2011**	0.45	8.86	<0.001
**2012**	0.35	6.55	<0.001

### Relationship between HFMD incidence and climatic indicators

On the basis of DIC (Table 3), Spatio-temporal model and Spatial model were quite similar and superior to Non-spatial model, indicating that spatial heterogeneity existed, while temporal effect can be ignored. However, Spatio-temporal interactive model including spatio-temporal interaction had the lowest DIC. Thus, further analysis should be focused on Spatio-temporal interactive model.

**Table 3:** **The deviance information criterion (DIC) for Bayes models**

**Model specifications**	**DIC**
**Non-spatial model**	138655.00
**Spatial model**	81210.60
**Spatio-temporal model**	81215.80
**Spatio-temporal interactive model**	6938.09

In Table 4, the parameter estimates for association and the corresponding confidence interval (CI) were presented. The results indicated that all variables except annual precipitation were significantly positive related to the risk of HFMD. Among them, annual average pressure was most important with parameter estimate 0.1054 (95% CI: 0.1014 to 0.1078), followed by annual average temperature with parameter estimate 0.0956 (95% CI: 0.08272 to 0.1076).

**Table 4:** **Estimated the effects of climatic indicators on HFMD by Spatio-temporal interactive model fitted, Shandong Province, China, 2008-2012**

**Covariates**	***β***	**95% confidence interval**
**Intercept**	−28.7500	−29.0100, −28.4100
**Average temperature**	0.0956	0.0827, 0.1076
**Annual average pressure**	0.1054	0.1014, 0.1078
**Average relative humidity**	0.0741	0.0551, 0.1034
**Average wind speed**	0.0705	0.0198, 0.1267
**Annual sunshine hours**	0.0557	0.0049, 0.1043
**Annual precipitation**	0.0473	−0.0125, 0.1393

**Figure 3:**
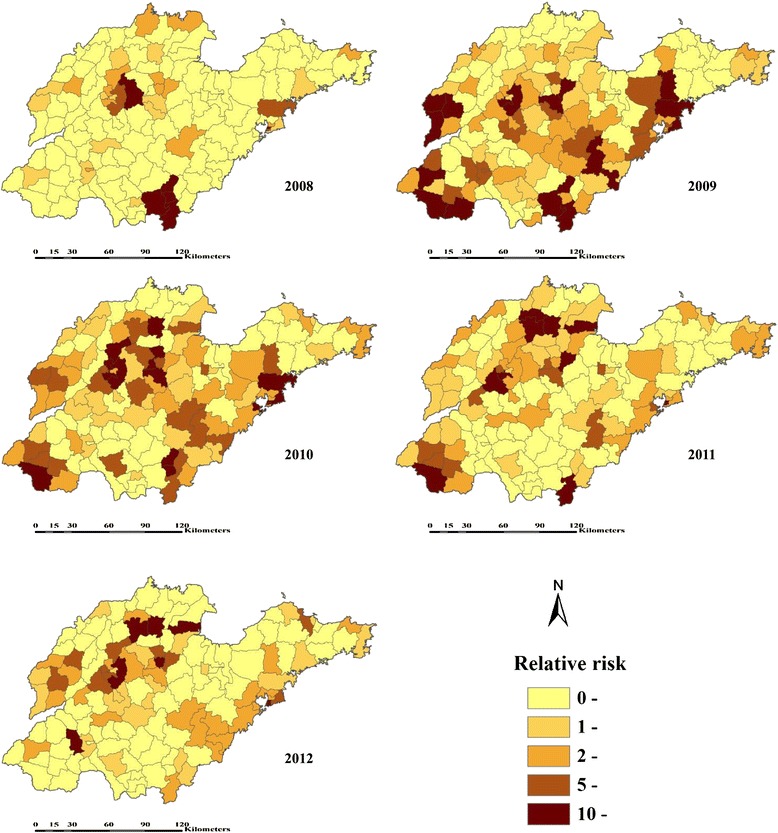
**Estimated relative risk for HFMD by Bayesian Spatio-temporal interactive model across 140 counties in Shandong Province, 2008–2012.**

Furthermore, the neighbourhood *RR* ranged from 0.0072 to 29.6827 for 2008, 0.0139 to 37.7516 for 2009, 0.0168 to 63.6974 for 2010, 0.0162 to 58.7585 for 2011, and 0.0634 to 30.4432 for 2012. Figure 3 displayed the spatial distribution of the estimated *RR*, where 36, 87, 91, 79, 65 out of 140 counties for 2008–2012 have *RR* greater than 1.

## Discussion

Shandong Province had been one of the most serious HFMD epidemic areas in China, with annual average incidence rate 104.40 per 100,000 during 2008 to 2012. Most HFMD cases (95.04%) were aged less than 6 years old, with an average male-to-female sex ratio 1.69 (see Table 1). Single seasonal peaks except double peaks in 2009, could be found between April and August during the study period (see Figure 2), which were different from other districts of China. For example, in Jiangsu Province, double peaks (the highest occurrence between April and June and the second occurring in November) appeared [45]; in Hong Kong, warmer seasonal peak (May-July) and winter peak (October-December) were detected [17]; in Guangdong Province, HFMD incidence peaked in April-May and September-October [19]. These difference might be partly due to some risk factors, such as climatic, geographic and social factors [15,19]. In addition, spatial autocorrelation test indicated that HFMD had positive spatial autocorrelation at county level in Shandong Province (Table 2). This also confirmed that the occurrence of HFMD might be closely related to certain ecological factors in the specific area.

Considering the goodness-of-fit by the DIC, the model including the spatial and temporal interactive effects was best fitted (DIC =6938.09, Table 3), which demonstrated a strong spatio-temporal heterogeneity in HFMD risk at the county scale, with clusters of high risk areas, as previously reported [27]. And the HFMD incidence rate was correlated with annual average temperature, annual average pressure, annual average relative humidity, annual average wind speed, and annual sunshine hours (Table 4), which were partly compatible with previous ecological analyses from the literature [20,22,46], inferring that HFMD continued to be a disease related to climate. In this study, much more precise estimates of *RR* were obtained from Spatio-temporal interactive model, confirming the hypothesis that the risk of the disease was related to what was occurring in the neighbourhood of each spatial unit. Figure 3 clearly displayed the spatio-temporal characteristic of *RR*, the epidemic of HFMD extended continuously over time, and the spatial pattern changed from concentrating on minority counties to expanding to more areas (especially in 2009 and 2010). Also, many high risk counties were located in areas for which HFMD clusters have been detected [27]. Certainly, these climatic factors are hard to change, while adaptive and objective prevention and control mechanism can be established according to the active detected possible impacts, e,g., advanced prevention for high risk areas.

Bayesian spatio-temporal interactive model was a valuable tool for the spatial and temporal interactive assessment of disease patterns that could help to identify county differences, and explore possible risk factors simultaneously. This method could solve most of the problems faced by traditional statistical methods, such as the spatial autocorrelation and the potential dependence between the covariates [47]. In previous studies, Scan statistics methods had been used to determine the spatial or spatio-temporal distribution of HFMD [23,27-30]. These approaches might absorb the surrounding regions and generate false-positives areas due to a lack of specificity, that is, some non-cluster areas might be determined to be clustered since they encompassed many neighbourhoods and tended to detect larger clusters than expected [48,49]. However, the scan statistics could be complementary to Bayesian method. That was to say, the scan statistic detected general regions in which the risk was significantly high and the Bayesian posterior distribution further helped to identify the neighbourhoods contributing strongly to the scan statistic circle [50]. Thus, results for cluster analysis should be interpreted with knowledge of the spatial rate distribution, such as spatial Bayesian rates in particular [51]. Furthermore, the analysis unit (i.e., county) was also considered adequately given the disease and risk factors information that was available and the spatial level at which policies were taken. However, one must note that ecologic bias was inevitable in any ecological study [52]. In addition, some results might be biased due to the artificial grouping of observations and variables at the county level. Despite these limitations, this approach was very useful to explore spatially aggregated data and to highlight the most risky areas to conduct more accurate analysis.

## Conclusions

Using a Bayesian approach to estimate the contribution of climatic indicators on the spatial-temporal pattern of HFMD should be encouraged in epidemiology. Our results confirmed the spatial-temporal heterogeneity of HFMD distribution, with high risk in particular areas observed in Shandong Province, and the importance of climatic covariate. The results may help public health authorities to set up priorities regarding to be targeted for prevention or control measures.

## Abbreviations

HFMD: Hand, foot, and mouth disease
CISDCP: China Information System for Disease Control and Prevention
*RR*: Relative risk
DIC: Deviance Information Criterion
CI: Confidence interval
